# Clinical Significance of the Expression of Co-Stimulatory Molecule B7-H3 in Papillary Thyroid Carcinoma

**DOI:** 10.3389/fcell.2022.819236

**Published:** 2022-04-12

**Authors:** Bohui Zhao, Zehao Huang, Xinyi Zhu, Huizhu Cai, Yingcheng Huang, Xiwei Zhang, Zongmin Zhang, Haizhen Lu, Changming An, Lijuan Niu, Zhengjiang Li

**Affiliations:** ^1^ Department of Head and Neck Surgery, National Cancer Center/National Clinical Research Center for Cancer/Cancer Hospital and Shenzhen Hospital, Chinese Academy of Medical Sciences and Peking Union Medical College, Shenzhen, China; ^2^ Department of Head and Neck Surgery, National Cancer Center/National Clinical Research Center for Cancer/Cancer Hospital, Chinese Academy of Medical Sciences and Peking Union Medical College, Beijing, China; ^3^ Department of Pathology, National Cancer Center/National Clinical Research Center for Cancer/Cancer Hospital, Chinese Academy of Medical Sciences and Peking Union Medical College, Beijing, China; ^4^ Department of Ultrasound, National Cancer Center/National Clinical Research Center for Cancer/Cancer Hospital, Chinese Academy of Medical Sciences and Peking Union Medical College, Beijing, China

**Keywords:** papillary thyroid cancer, B7-H3, immune checkpoint, immunotherapy, metastasis, recurrence

## Abstract

**Background:** B7-H3, also known as CD276, an important immune checkpoint member of the B7-CD28 family, is confirmed as a promising target after PD-L1 in clinical trials. Although the overexpression of B7-H3 has been associated with invasive metastatic potential and poor prognosis in multiple types of cancer, nothing is known regarding the expression profiles of B7-H3 in papillary thyroid carcinoma (PTC). In this study, we carried out a large-scale analysis of B7-H3 expression in PTC patients and evaluated the potential clinical significance of B7-H3.

**Methods:** In total, data from 1,210 samples, including 867 cases from TCGA and four GEO datasets, were collected for B7-H3–related transcriptome analyses, and 343 postoperative, whole-tumor sections were collected from patients with PTC at our institute for B7-H3–specific immunohistochemistry (IHC) staining. The statistical analysis was primarily accomplished using the R project for statistical computing.

**Results:** B7-H3 positivity was found in 84.8% of PTC patients (291/343), and the mRNA and protein expression levels of B7-H3 in PTC were markedly higher than those of para-tumor tissues (*p* < 0.001), demonstrating that B7-H3 can serve as a potential diagnostic biomarker for PTC. The significant upregulation of B7-H3 in PTC is caused by distinct patterns of CNVs and CpG DNA methylation. Functional enrichment analysis confirmed that high B7-H3 expression was significantly associated with specific immune features and angiogenesis. High B7-H3 protein expression was associated with tumor size (*p* = 0.022), extrathyroidal extension (ETE) (*p* = 0.003), and lymph node metastasis (LNM) (*p* < 0.001). More importantly, multivariate analysis confirmed that B7-H3 was an independent predictor of relapse-free survival (RFS) (*p* < 0.05). In the subgroup analysis, positive B7-H3 staining was associated with worse RFS in patients with primary tumor size ≥2 cm (*p* < 0.05), age ≥55 years (*p* < 0.05), LNM (*p* = 0.07), multifocality (*p* < 0.05), and ETE (*p* < 0.05). In addition, Circos plots indicated that B7-H3 was significantly associated with other immune checkpoints in the B7-CD28 family.

**Conclusion:** This is the first comprehensive study to elucidate the expression profile of B7-H3 in PTC. Our observations revealed that B7-H3 is a novel independent biomarker for predicting LNM and disease recurrence for PTC patients, and it thus may serve as an indicator that could be used to improve risk-adapted therapeutic strategies and a novel target for immunotherapy strategies for patients who undergo an aggressive disease course.

## Introduction

In recent years, encouraging developments have been reported for patients undergoing antibody treatments against immune checkpoints in the B7-CD28 family, such as CTLA4 and PD-1/PD-L1, which have brought about revolutionary changes in cancer treatment (Gong et al., 2018; Andrews et al., 2019). These achievements piqued our interest regarding whether immune checkpoints could be exploited as therapeutic targets for papillary thyroid carcinoma (PTC) treatment. Therefore, we conducted an exploration of public databases containing data regarding B7-CD28 family members in PTC patients.

B7-H3, known alternatively as CD276, belongs to the B7-CD28 immunoregulatory protein superfamily (Flem-Karlsen et al., 2018). Although the identities of its binding partners remain unclear, a wide range of B7-H3 expression has been reported in multiple tumor types, including non–small cell lung cancer (Carvajal-Hausdorf et al., 2019a), prostate cancer (Yuan et al., 2011), and renal cell cancer (Crispen et al., 2008). Although B7-H3 was initially characterized as a T cell co-stimulating protein, most recent studies have reported that B7-H3 is a T cell inhibitor that promotes tumor aggressiveness and proliferation (Picarda et al., 2016; Flem-Karlsen et al., 2018). Critically, B7-H3 seems to play vital roles in tumor growth and metastasis, and B7-H3 expression is associated with poor prognosis (Tekle et al., 2012). However, reports regarding B7-H3 expression in PTC are lacking; therefore, in this study, we performed a comprehensive analysis of B7-H3 expression in PTC.

## Materials and Methods

### Publicly Available Data

We collected data for 867 PTC cases from five public datasets culled from The Cancer Genome Atlas (TCGA) (https://genomecancer.ucsc.edu) and Gene Expression Omnibus (GEO) (http://www.ncbi.nlm.nih.gov/geo), including 555 RNA-seq datasets (Illumina HiSeq 2000) from TCGA, 40 microarray cases from GSE29265, 95 microarray cases from GSE33630, 116 microarray cases from GSE35570, and 61 microarray cases from GSE60542. The microarray datasets from the GEO were log2-transformed and quantile-normalized prior to the analysis.

### Patients and Samples

To verify the discoveries made by studying the public databases listed above, 343 patients with PTC who underwent surgery from 2003 to 2018 at the Department of Head and Neck Surgery, National Cancer Center, were included in this study. This study was approved by the Ethics Committee of the National Cancer Center/Cancer Hospital, Chinese Academy of Medical Sciences. Informed consent requirements were waived due to the retrospective nature of this study, and all data were analyzed anonymously.

All 343 eligible patients were pathologically confirmed as PTC patients. All histological slides were independently re-reviewed according to the current WHO criteria by pathologists who were blind to disease outcomes. The data for 343 patients were extracted from the Department of Medical Informatics database to ensure the availability of an adequate medical history. At the time of diagnosis, all patients enrolled in this study had no history of previous thyroid surgery. The subjects with other types of malignancies or other histological types of thyroid cancer, for example, anaplastic thyroid cancer were systematically excluded.

### Patient Characteristics and Clinical Outcomes

The clinicopathological variables of all patients, including sex, age at time of diagnosis, maximum tumor size, multifocal lesions, extrathyroidal extension (ETE), and lymph node metastasis (LNM), were evaluated comprehensively. TNM staging was classified based on the American Joint Committee on Cancer (AJCC, eighth edition) criteria for differentiated thyroid cancer. RFS was specified as the period from the date of first surgery to the date of recurrence, metastasis, or the time of last censoring. Recurrence was considered the appearance of disease proven by biopsy/confirmed by secondary surgery.

The clinicopathological features of 343 PTC patients are summarized in Table 1; their median age at the time of surgery was 47 years and ranged from 12 to 67. The majority of subjects were female (76%). Lobectomy and total thyroidectomy (TT) were performed for 161 (46.9%) and 182 (53.1%) patients, respectively, as the initial surgical treatment for the primary tumor. Central lymph node dissection (CLND) was performed for every patient, and lateral lymph node dissection (LLND) was performed for 132 (38.5%) patients. According to the final pathological evaluations, 175 (51.0%) patients with LNM were identified among all patients in the study. No patients had a history of undergoing head and neck radiation treatment or surgery at the initial diagnosis. The mean follow-up duration was 121 months (ranging from 13 to 161 months). Upon conclusion of the follow-up evaluation, 26 subjects suffered recurrence of the disease, but none died from PTC as the specific cause.

**TABLE 1:** Relationship of B7-H3 expression by clinicopathological factors in PTC.

Characteristics	Total (N = 343)	B7-H3 expression in PTCs		
		Negative (N = 132, 38.5%)	Positive (N = 211, 61.5%)	*p*-value
Sex, n (%)				0.111
Male	78 (22.7)	24 (18.2)	54 (25.6)	
Female	265 (77.3)	108 (81.8)	157 (74.4)	
Age, y, n (%)				0.361
<55	286 (83.4)	107 (81.1)	179 (84.8)	
≥55	57 (16.6)	25 (18.9)	32 (15.2)	
Multifocality, n (%)				0.88
Yes	116 (33.8)	44 (33.3)	72 (34.1)	
No	227 (66.2)	88 (66.7)	139 (65.9)	
Extrathyroidal extension, n (%)				0.003
Yes	215 (62.7)	70 (53.0)	145 (68.7)	
No	128 (37.3)	62 (47.0)	66 (31.3)	
Lymph node metastasis, n (%)				<0.001
Yes	175 (51.0)	39 (29.5)	136 (64.5)	
No	168 (49.0)	93 (70.5)	75 (35.5)	
Recurrence, n (%)				0.001
Yes	27 (7.9)	2 (1.5)	25 (11.8)	
No	316 (92.1)	130 (98.5)	186 (88.2)	
Hashimoto thyroiditis, n (%)				0.018
Yes	57 (16.6)	14 (10.6)	43 (20.4)	
No	286 (83.4)	118 (89.4)	168 (79.6)	
Length, cm, n (%)				
<1	143 (41.7)	68 (51.5)	75 (35.6)	0.022
1–2	159 (46.4)	53 (40.2)	106 (50.2)	
2–4	34 (9.9)	10 (7.6)	24 (11.4)	
>4	7 (2.0)	1 (0.7)	6 (2.8)	
T stage, n (%)				0.028
I	29 (8.5)	15 (11.4)	14 (6.6)	
II	49 (14.3)	23 (17.4)	26 (12.3)	
III	69 (20.1)	44 (33.3)	25 (11.9)	
IV	196 (57.1)	50 (37.9)	146 (69.2)	

### Initial Treatment

All patients underwent preoperational ultrasonography. The patients with suspected lateral LNM underwent additional CT scans. Lobectomy and TT were selected for each patient based on the condition of the primary tumor. All the subjects received CLND. Modified lateral lymph node dissection included levels II-IV as the minimal range. Intraoperative frozen section (FS) histology was used to aid surgeons in determining the scope of surgical plans. All the patients underwent postoperative thyroid-stimulating hormone (TSH) suppressive treatment.

### Immunohistochemistry

Immunohistochemistry (IHC) staining for B7-H3 was interpreted by two pathologists in a blinded manner. Representative 4-μm-thick, formalin-fixed, paraffin-embedded (FFPE), resected specimens were placed onto the glass slides after deparaffinization, rehydration, antigen retrieval, endogenous peroxidase inactivation, and blocking of nonspecific binding. B7-H3 was stained overnight at 4°C using the D9M2l rabbit monoclonal antibody (1:200, clone D9M2l, catalog 14058s, Cell Signaling Technology, Danvers, MA, United States ). Finally, the reaction products were imaged using a DAKO EnVision Detection System (Dako), and hematoxylin was used for counterstaining.

### Quantification of B7-H3 Expression

The B7-H3 staining intensity was scored as follows: negative (–), faint/weak (+), medium (++), or strong (+++). The staining extent was scored according to the percentage of positively stained areas in tumor cells (TCs). The staining intensity and percentage positivity scores for TCs were then multiplied to compute the immunoreactivity score. The final B7-H3 staining pattern was scored as 0, 1, 2, and 3 in the following manner: 0, no membranous staining or <1% TCs with faint/weak membranous staining; 1, ≥1% TCs with faint/weak membranous staining or <1% TCs with medium membranous staining; 2, ≥1% TCs with medium membranous staining or <1% TCs with strong membranous staining; and 3, ≥1% tumor cells with strong membranous staining. The tissue samples with a final staining score of 0 or 1 were assigned to the B7-H3–negative group, while the specimens with a final staining score of 2 or 3 were assigned to the B7-H3–positive group.

### Statistical Analysis

All statistical analyses and figure generation were carried out in SPSS version 25.0, GraphPad Prism version 8.0, and R version 3.5.1. Chi-square tests or Fisher’s exact tests were used to assess the relationships between B7-H3 status and clinicopathological characteristics. The Mann–Whitney *U*-test was used to calculate the distribution of B7-H3 in different groups. The difference between the survival rates of the B7-H3–positive and B7-H3–negative groups was assessed using the Kaplan–Meier method with a log-rank test. Additional figures were created in the R statistical software environment using several graphics packages, including *ggplot2*, *pROC*, and *survival*. The threshold for statistical significance was *p* < 0.05 for all statistical methods.

## Results

### B7-H3 Is Overexpressed in PTC Tumors

**FIGURE 1:**
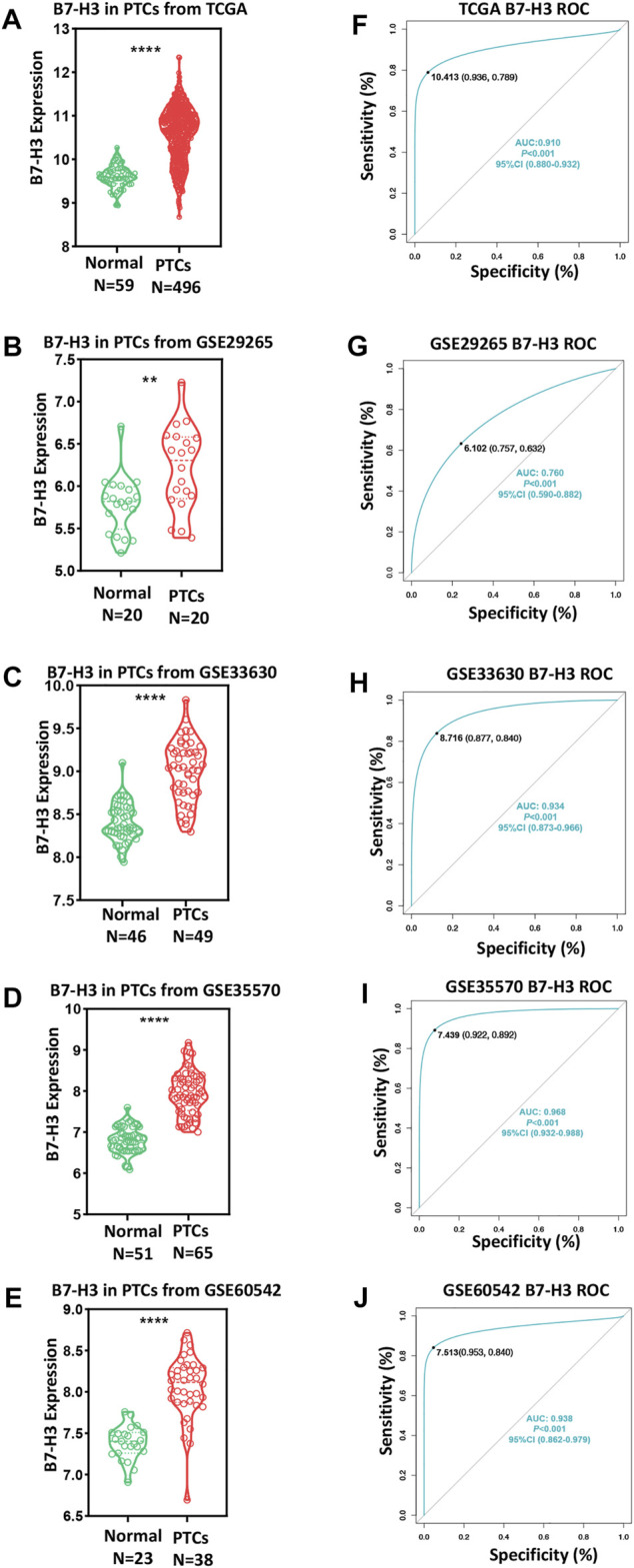
B7-H3 expression of PTC and para-tumor tissue in TCGA and GEO datasets **(A–E)**. ROC curve of B7-H3 expression in TCGA and GEO datasets **(F–J)**. ** and **** represent *p* < 0.01 and *p* < 0.0001, respectively.

To investigate the role of B7-CD28 family members in PTC, we assessed TCGA datasets to analyze the mRNA levels of transcripts encoding B7-CD28 family member proteins. The results showed that the B7-H3 mRNA level was higher than that of any other member of the B7-CD28 family in PTC (Supplementary Figure S1), indicating that B7-H3 may play a preponderant role in the development of PTC. We next determined the B7-H3 expression profiles of PTC tumors and para-tumor tissue samples from 555 patients. The results from both TCGA and GEO datasets showed that expression of B7-H3 was significantly upregulated in PTC tumors in comparison with para-tumor tissue (Figures 1A–E). ROC analysis was then used to investigate the potential diagnostic value of B7-H3 expression in PTC. The AUCs of B7-H3 in TCGA and four GEO datasets were 0.91, 0.76, 0.93, 0.97, and 0.94, which demonstrated aberrant B7-H3 expression, as we expected (Figures 1F–J). This finding prompted us to investigate the biological mechanism underlying abnormal B7-H3 expression in PTC tumors. Therefore, we performed further analysis based on TCGA datasets, which showed that the abnormal expression of B7-H3 in PTCs was closely related to distinct patterns of CNVs and CpG DNA methylation (Supplementary Figure S2).

### B7-H3 Expression and Clinicopathological Features

**FIGURE 2:**
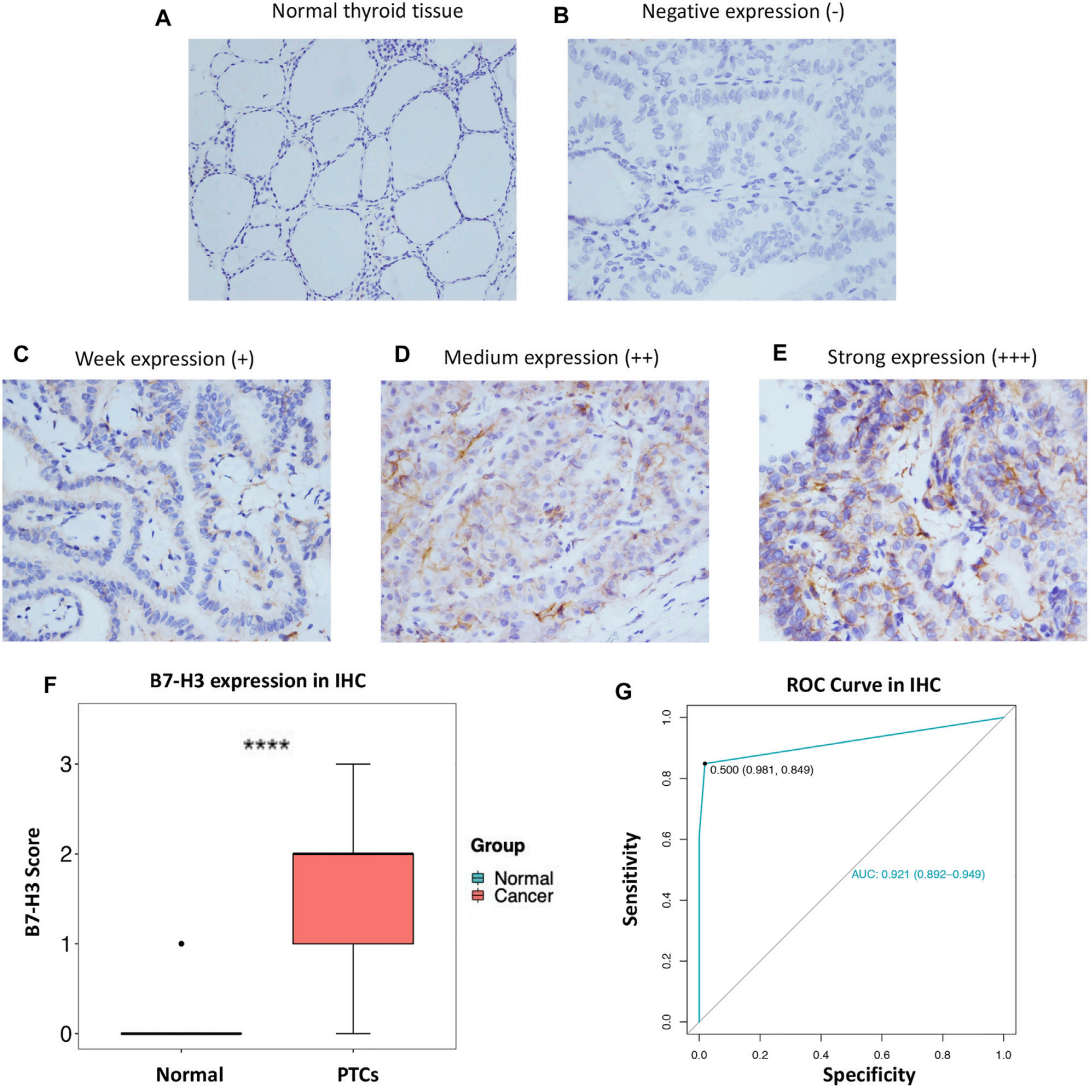
Protein expression profile of B7-H3 in PTC (200 ×) and normal thyroid tissue (100 ×) **(A–E)**. B7-H3 expression score in PTC and normal tissue **(F)**. ROC curve of B7-H3 protein expression in PTC **(G)**. **** represents ***p*** <0.0001.

In order to further explore our findings from TCGA, we examined the expression of B7-H3 by IHC in 343 PTC tumors and 159 para-tumor tissue samples. The results showed that B7-H3 staining in PTC TCs was mainly located in the cell membrane, and the corresponding para-tumor tissues showed extremely weak staining. In contrast, B7-H3 expression was relatively high in PTC TCs; among the PTC TC samples, 24 samples were scored as 0 (15.1%), 38 samples were scored as 1 (23.9%), 68 samples were scored as 2 (42.8%), and 29 samples were scored as 3 (18.2%) (Figures 2A–E).

Overall, the B7-H3 expression levels of nontumor tissue samples were significantly lower than those of their paired thyroid tumor tissue samples (*p <* 0.001) (Figure 2F). Next, we performed ROC analysis based on the IHC score of each sample. The results of the ROC analysis suggested that the B7-H3 protein level has diagnostic value for PTC patients (Figure 2G).

To further analyze the correlations between B7-H3 protein expression and clinical parameters, we classified IHC staining scores of 2 and 3 as positive B7-H3 staining, whereas scores of 0 and 1 were classified as negative B7-H3 staining. Using this method, B7-H3 expression was validated at the protein level for the total cohort of 343 specimens, among which 52 (15.2%) samples were scored as 0, 80 (23.3%) samples were scored as 1, 137 (39.9%) samples were scored as 2, and 74 (21.6%) samples were scored as 3. Therefore, 132 (38.5%) samples were assigned to the B7-H3–negative group and 211 (61.5%) samples were assigned to the B7-H3–positive group. The relationships between B7-H3 expression and clinical features are shown in Table 1. B7-H3 protein expression in PTC was found to be closely correlated with tumor length (*p* = 0.022), ETE (*p* = 0.003), LNM (*p* < 0.001), and recurrence (*p* = 0.001), but it was uncorrelated with sex and age (*p* > 0.05).

### B7-H3-Related Biological Processes and Pathway in PTCs

**FIGURE 3:**
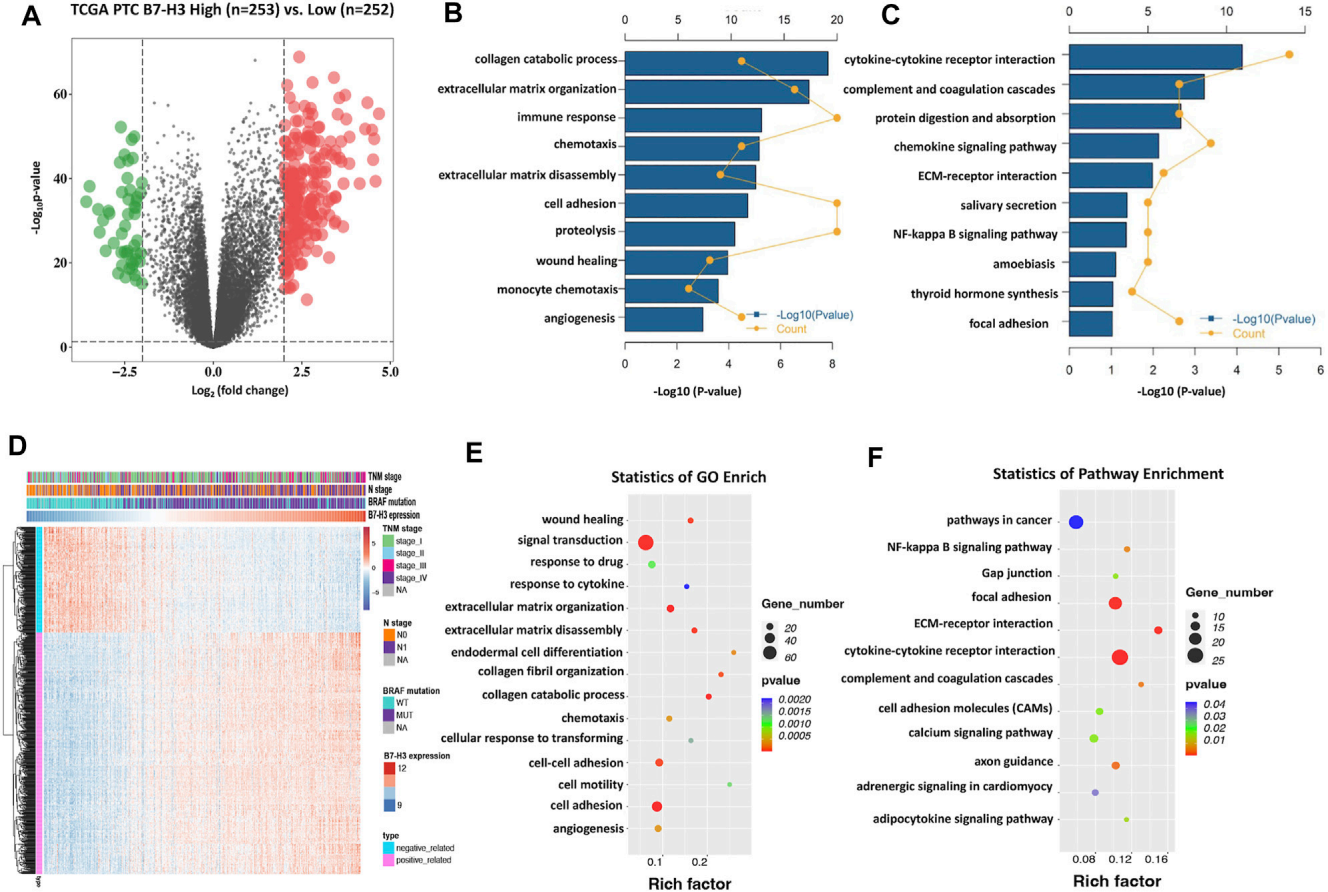
B7-H3–related biological process and pathways in PTC. Differentially expressed genes in Volcano plots from TCGA dataset **(A)**. Gene enrichment with GO terms of selected genes **(B)**. Gene enrichment with KEGG terms of selected genes **(C)**. Particulars of risk score and most-related genes in heat map **(D)**. The bubble plots showing that B7-H3 is closely correlated with cell invasion, immune response and angiogenesis **(E,F)**.

Given that B7-H3 expression was found to be significantly correlated with tumor aggressiveness, we explored the underlying mechanism linking this clinical feature with B7-H3 expression in PTCs. Here, two different methods were used to reveal the B7-H3–specific biological landscape in PTCs. First, based on the median expression level of B7-H3 from TCGA datasets, the test subjects were divided into two groups: a B7-H3 high-expression group (N = 253) and a B7-H3 low-expression group (N = 252). Next, we identified 266 differentially expressed genes (DEGs) between the B7-H3 high- and low-expression groups using the following criteria: fold-change > 4 and *p* < 0.0001 (Figure 3A). Finally, 216 upregulated and 50 downregulated 50 genes were obtained for the B7-H3 high group, after which Gene Ontology (GO) and Kyoto Encyclopedia of Genes and Genomes (KEGG) analyses were conducted based on these DEGs. The results of the GO and KEGG analyses showed that the selected DEGs were likely to be involved in the immune response, extracellular matrix disassembly, and angiogenesis (Figures B,C). Next, we selected the set of genes with the most significant correlation with B7-H3 expression in PTC TCs (|R| > 0.6, *p* < 0.0001) (Figures 3D–F), which included 508 positively related genes and 223 negatively related genes. GO and KEGG analyses of the selected gene sets showed that they were mainly enriched in cell adhesion–related pathways and participated in the process of extracellular matrix decomposition, cell motility, and angiogenesis, suggesting that B7-H3 expression may be associated with PTC metastasis.

### Prognostic Features of B7-H3 Expression in PTCs

**FIGURE 4:**
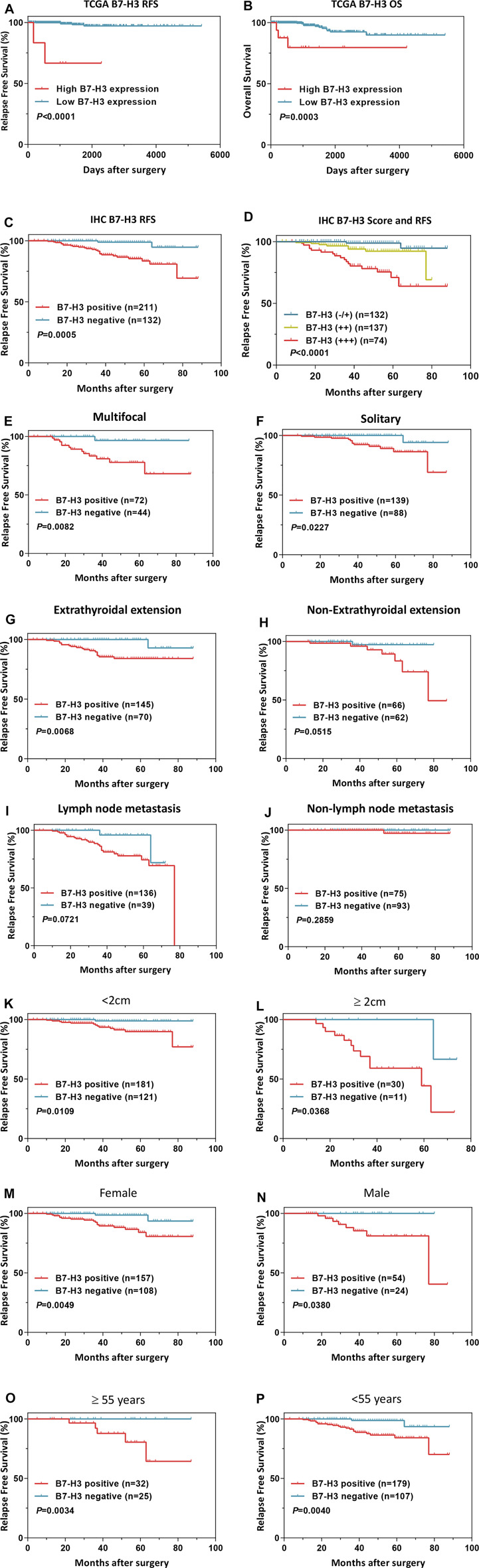
Survival analysis of B7-H3 in PTC of TCGA dataset **(A,B)**. B7-H3 survival analysis in PTC of IHC **(C,D)**. Survival analysis based on B7-H3 expression in clinical subgroups **(E–P)**. *p* < 0.05 is considered to be statistically significant., ,

Next, we assessed the association between B7-H3 expression and PTC prognosis. First, we performed a survival analysis using TCGA dataset. The results showed that the B7-H3–positive status was strongly associated with increased risks of relapse (Figure 4A, *p* < 0.001) and death (Figure 4B, *p* < 0.001) in comparison with B7-H3–negative status. Verification of these results at the protein level revealed that B7-H3–positive subjects showed dramatically worse RFS in comparison with that of B7-H3–negative subjects (Figure 4C, *p* < 0.001). Moreover, the analysis of patient prognosis suggested that the B7-H3 IHC staining score was positively correlated with poor prognosis (Figure 4D, *p* < 0.001). In addition, the B7-H3–related survival analysis was conducted in patient subgroups that were stratified using different clinicopathological variables. Using the Kaplan–Meier method and log-rank test, it was determined that B7-H3 positive staining was correlated with worse RFS in the following subgroups: tumor size ≥2 cm (*p* < 0.05), male (*p* < 0.05), age ≥55 years (*p* < 0.05), multifocality (*p* < 0.05), and ETE (*p* < 0.05) (Figures 4E–P).

Finally, we explored the utility of B7-H3 expression as an independent prognostic factor for PTC patients by conducting univariate and multivariate Cox regression analyses. The univariate analysis revealed that tumor length (*p* < 0.001), multifocality (*p* = 0.024), LNM (*p* < 0.001), and B7-H3 positivity in PTCs (*p* = 0.004) were significantly related to RFS, and the multivariate analysis demonstrated that these four characteristics were also independent predictors of RFS (Table 2, *p* < 0.05). The other selected variables were not significantly associated with PTC recurrence.

**TABLE 2:** Univariable and multivariable Cox regression analyses for RFS in PTC patients.

Variable		Univariate analysis	*p*-value	Multivariate analysis	*p*-value
		HR (95%CI)		HR (95%CI)	
Sex	Male/female	0.698 (0.305–1.596)	0.394	1.374 (0.545–3.465)	0.501
Age	≥55/<55	1.111 (0.420–2.934)	0.832	1.057 (0.332–3.368)	0.925
Length	≥2/<2	9.034 (4.176–19.540)	**<0.001**	5.617 (2.335–13.513)	**<0.001**
Multifocality	Yes/no	2.393 (1.125–5.094)	**0.024**	3.512 (1.517–8.132)	**0.003**
Extrathyroidal extension	Yes/no	1.364 (0.597–3.118)	0.461	1.145 (0.384–3.412)	0.807
Hashimoto thyroiditis	Yes/no	0.454 (0.107–1.927)	0.285	0.383 (0.085–1.737)	0.214
Lymph node metastasis	Yes/no	40.095 (5.307–302.900)	**<0.001**	25.842 (3.015–221.522)	**0.003**
B7-H3	Positive/negative	8.369 (1.981–35.351)	**0.004**	4.566 (1.056–19.744)	**0.042**

*p*-value < 0.05 in bold are statistically significant. Abbreviations: HR, hazard ratio; CI, confidence interval; RFS, recurrence-free survival; PTC, papillary thyroid carcinoma.

### The Relationship Between B7-H3 and B7-CD28 Family Checkpoint Members

**FIGURE 5:**
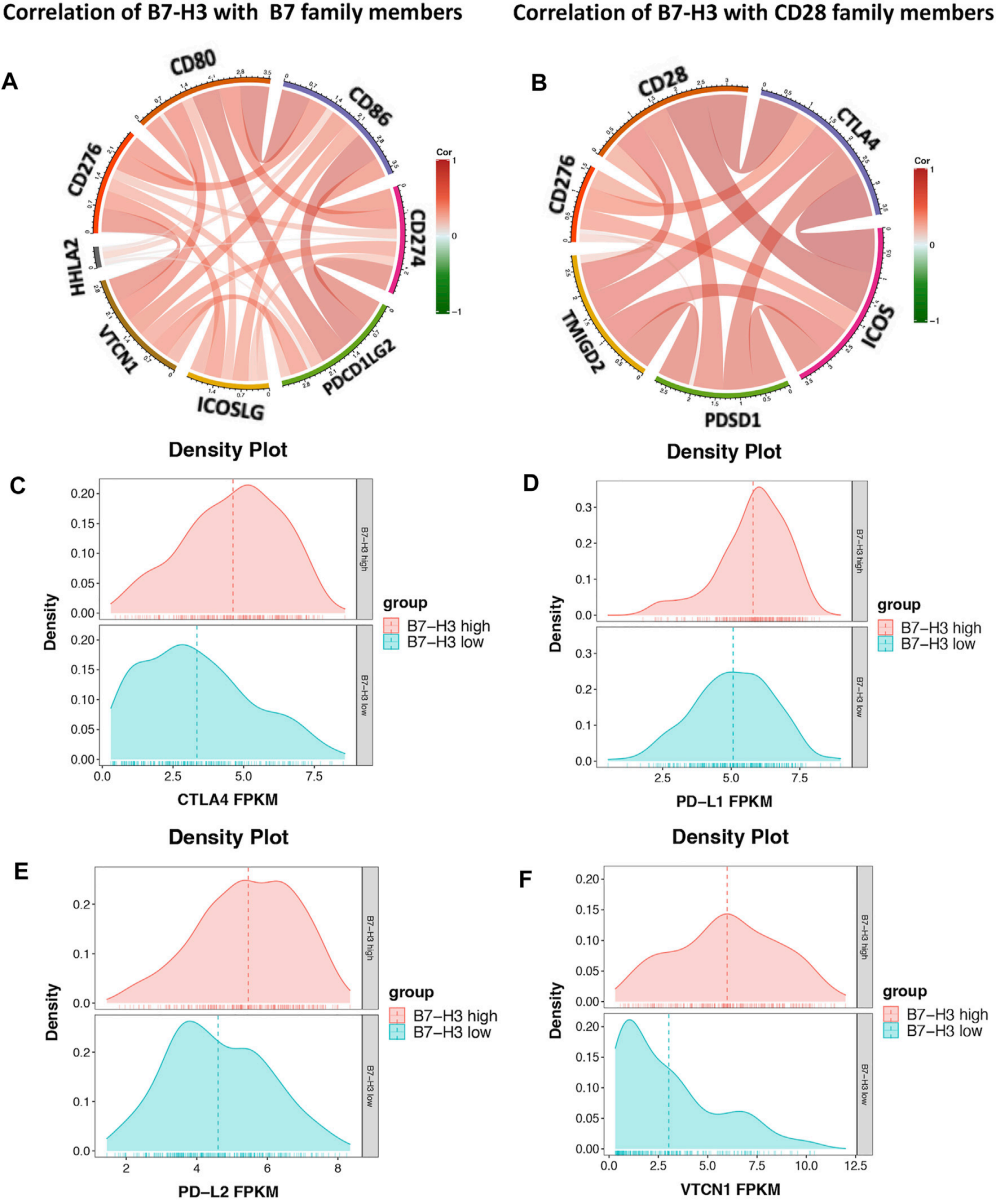
Correlation of B7-H3 and B7 family members **(A)**. Correlation of B7-H3 and CD28 family members **(B)**. Distribution of PD-L1, PD-L2, CTLA4, and VTCN1 in high- and low-expression groups of B7-H3 **(C–F)**.

The results described above suggest that B7-H3 is a promising target for PTC treatment. The relationship between B7-H3 and B7-CD28 family checkpoints was assessed using TCGA datasets. The results showed that B7-H3 expression was significantly correlated with the expression levels of most B7-CD28 family members, and the correlations with the expression levels of PD-L1, PD-L2, CTLA4, and VTCN1 were particularly strong. These findings suggest that these markers may exert synergistic effects in the context of PTC, indicating that multicheckpoint blockade strategies may be promising treatment methods for PTC patients (Figure 5).

## Discussion

Given the high risk of metastasis and relapse associated with PTC and the limited available treatment options and lack of biomarkers for identifying potential high-risk patients, novel treatment strategies are urgently needed for the patients afflicted with this disease. The recent widespread success of immunotherapy methods targeting the B7-CD28 family members in a variety of malignancies, specifically the immune checkpoint B7-H3, has engendered hope that these strategies could also be applied to other types of cancer, including PTC. Although recent studies suggest that B7-H3 shows significant potential as a treatment target for patients with several different types of solid malignancies and aberrant B7-H3 expression has been documented in various human cancers (Benzon et al., 2017; Nehama et al., 2019; Carvajal-Hausdorf et al., 2019b; Cai et al., 2020),^,^ a comprehensive analysis of B7-H3 expression in PTC had not been performed prior to this study. Herein, to explore B7-H3 expression profiles in PTC patients, data from a total of 1,210 samples, including 867 cases from TCGA and four GEO datasets, were collected for B7-H3–related transcriptome analyses, and 343 postoperative, whole-tumor sections were obtained from PTC patients at our institute for B7-H3–specific IHC staining.

Our analysis of TCGA and GEO datasets revealed that B7-H3 expression in PTC was conspicuously higher than that of other B7-CD28 family members, suggesting that targeting B7-H3 could be a more effective treatment strategy in comparison with targeting other B7-CD28 family members. Previous studies reported positive expression rates of B7-H3 in other human tumor tissues ranging from 37 to 76% due to the use of different scoring systems and antibodies for B7-H3 detection (Sun et al., 2006; Cheng et al., 2018; Inamura et al., 2018; Nehama et al., 2019; Kim et al., 2020). In our study, we found that B7-H3 expression was upregulated in the PTC tissues at the protein and mRNA levels, and IHC staining revealed B7-H3 protein expression in **most** of the PTC simples. In addition, our results revealed that abnormal B7-H3 expression in PTCs was closely correlated with distinct patterns of CNVs and CpG DNA methylation. These findings provide a potential mechanistic link between CNV/DNA methylation and increased expression of B7-H3, and they provide hope that the treatment strategies targeting DNA methylation regulators in combination with the administration of anti–B7-H3 monoclonal antibodies may exhibit superior curative effects for PTC patients in comparison with the currently available methods.

Given that B7-H3 protein was highly expressed on TCs and tumor-infiltrating blood vessels, while its expression level in normal human tissue was low, therapeutic strategies targeting B7-H3 may be able to achieve high therapeutic efficacy against cancer cells together with low toxicity in normal tissues (Seaman et al., 2007; Cheng et al., 2018). Our GO and KEGG analyses showed that the B7-H3–related gene sets were enriched in genes related to angiogenesis and cell motility. Therefore, we speculated that high B7-H3 expression may be related to PTC lymph node metastasis and poor patient prognosis (Nilsson and Heymach, 2006; Zeng et al., 2018). Our IHC results confirmed that B7-H3 expression was associated with tumor size, ETE, LNM, and recurrence, indicating that B7-H3 plays specific roles in tumor invasion and controlling the tumor microenvironment. Many reports have shown that B7-H3 expression is related to poor prognosis in various tumors (Picarda et al., 2016; Flem-Karlsen et al., 2018). In this study, we found that high B7-H3 expression was correlated with shorter RFS in PTC patients. The results of our survival analyses using TCGA datasets and IHC staining show that positive B7-H3 expression status is closely associated with poor prognosis for PTC patients. To verify this result, we explored the relationship between B7-H3 expression status and patient prognosis for subgroups stratified by age, ETE, LNM, and other features. Interestingly, although the B7-H3 expression status was correlated with patient prognosis for most of the subgroups, B7-H3 expression was not significantly associated with differences in RFS for the LNM and nonmetastasis subgroups (*p* = 0.0721). It is possible that no statistically significant relationship between RFS and B7-H3 expression was identified in this comparison because the B7-H3 expression levels of both groups were relatively high, and few subjects had low B7-H3 expression levels. More importantly, multivariate analysis was used to confirm that B7-H3 can serve as an independent prognostic factor for shorter RFS. These findings give us confidence that regardless of clinical features, B7-H3 can be used as a stable and effective prognosticator for identifying high-risk PTC patients.

Many studies have shown that B7-H3 predominantly acts as a molecular co-inhibitor at tumor sites to generate an immunosuppressive microenvironment (Suh et al., 2003; Zhang et al., 2018). The results of our study demonstrate that the expression of B7-H3 is closely related to that of most other B7-CD28 family members in PTC TCs. Promising results have been achieved with checkpoint inhibitors such as nivolumab and ipilimumab, resulting in U.S. FDA approval and widespread use in clinical settings (French et al., 2017; Larkin et al., 2019). Although the antibodies against B7-H3 are not currently used in clinical settings, the significant prognostic value of B7-H3 suggests that anti–B7-H3 monoclonal antibodies could provide significant benefits for PTC patients, especially those with advanced and iodine-resistant tumors.

In summary, we performed large-scale analyses exploring the clinical and immune features of B7-H3 in PTC patients. Our results demonstrate that the B7-H3 status of PTC TCs may serve as a predictive biomarker and facilitate the improvement of risk-adapted therapeutic strategies. However, the present analysis is limited by the potential effects of noise on our results and conclusions, so additional experiments should be performed to validate our findings.

## Data Availability

The datasets presented in this study can be found in online repositories. The names of the repository/repositories and accession number(s) can be found in the article/Supplementary Material. The other data used and/or analyzed during the current study are available from the corresponding author via reasonable request.
