# Supplementary figures and images for: Clinical Significance of the Expression of Co-Stimulatory Molecule B7-H3 in Papillary Thyroid Carcinoma
Source: Front Cell Dev Biol. 2022 Apr 12;10:819236. doi: 10.3389/fcell.2022.819236 (PMC9039293; doi:10.3389/fcell.2022.819236)

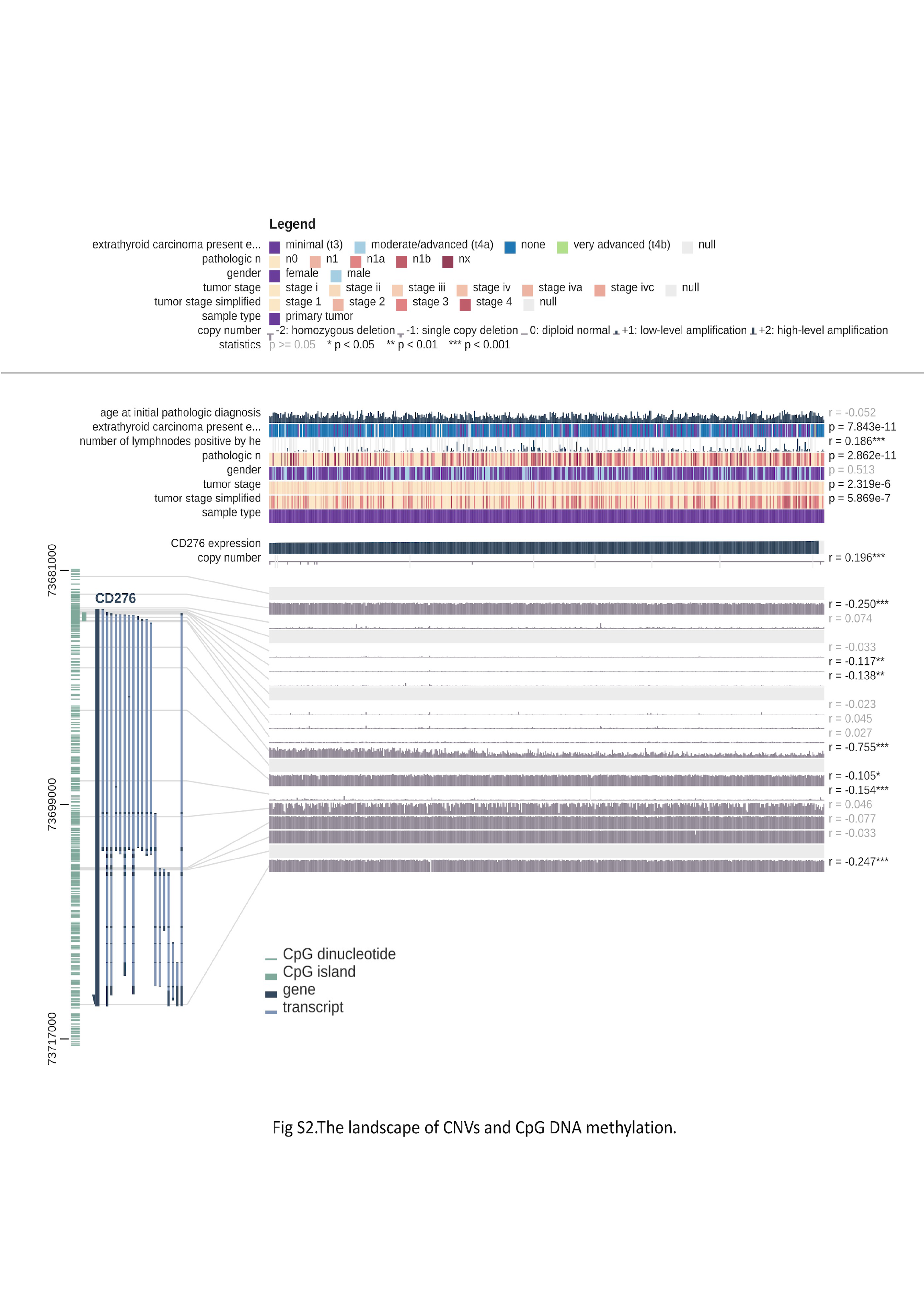

Supplement: Supplementary file 1 [file Image2.TIF]

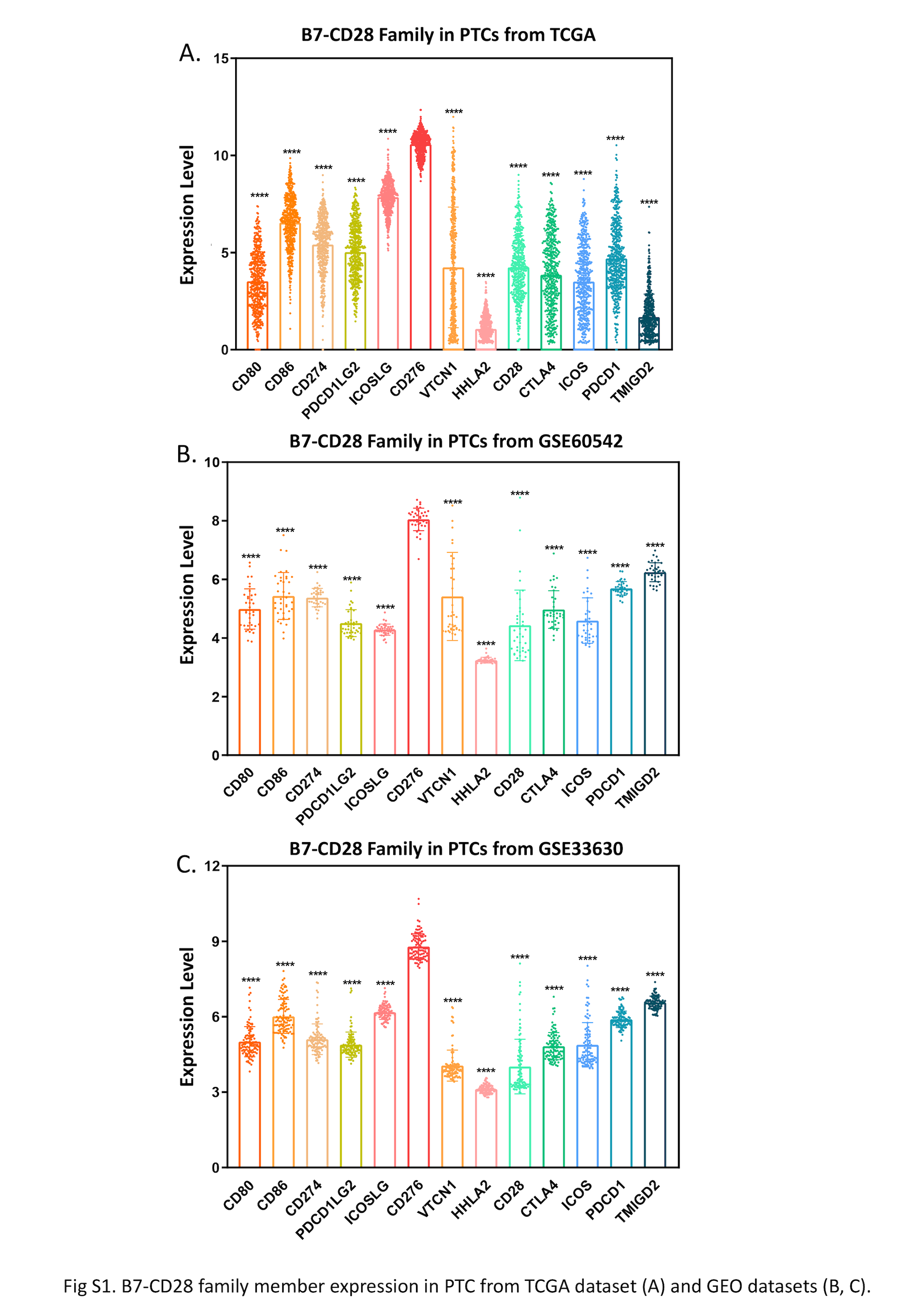

Supplement: Supplementary file 2 [file Image1.TIF]
